# Psychological Intervention in the Oocyte Pick-up Room and Recovery Room in Assisted Reproduction: new listening accounts

**DOI:** 10.5935/1518-0557.20190092

**Published:** 2020

**Authors:** Marcia Christina Gonçalves Gusmão, Lohayne Marins Teixeira, Ana Cristina Allemand Mancebo, Marcelo Marinho de Souza, Roberto de Azevedo Antunes, Maria do Carmo Borges de Souza

**Affiliations:** 1 Fertipraxis – Rio de Janeiro, RJ, Brazil; 2 Fertipraxis/Hospital Federal da Lagoa – Rio de Janeiro, RJ, Brazil

**Keywords:** psychology, acting out, IVF/ICSI, assisted reproduction

## Abstract

**Objective:**

This study seeks to identify the role and possible participation of a psychologist/psychoanalyst inside an Oocyte Pick-up Room and Recovery Room (OPR-RR) in an Assisted Reproduction clinic and its implications on patients and team.

**Methods:**

Prospective study of psychological support during the procedures from September 2014 to December 2018. Most visits took place during oocyte retrievals, for either IVF/ICSI or gamete freezing.

**Results:**

Of the 2,343 cases, the psychologist was present in 965 of them (41%), during oocyte retrievals, with available professionals in 59% of the times (722 cases). The embryo transfers (1,011) had psychological assistance in 20% of the time (218 cases). The intrauterine insemination cases were excluded for not happening in a surgical environment. The recovery room was identified as one of the spaces for welcoming and listening to anxieties, desires, projects, worries, fears, frustrations, joys and expectations for those who come to the clinic seeking the desire to gestate. The patients’ talks, collected in observations transcribed from what was heard, with dates and types of procedures, were discussed with either the team or the assistant physician. The team stands positively in the presence of a psychologist/psychoanalyst, who brings new perceptions and the development of the whole art of listening, for all involved.

**Conclusion:**

The presence of a psychologist/psychoanalyst in the Oocyte Pick-up Room and the Recovery Room in an Assisted Reproduction clinic means an opportunity to listen to patients’ emotions, providing well-being to patients and echoing in the teamwork relationships.

## INTRODUCTION

This study seeks to reflect on the construction of a clinical practice with a psychologist/psychoanalyst as part of a multidisciplinary team, at an Assisted Reproduction clinic. An “extramural” clinical practice, as Mezan (2002) cites, that is, beyond the office boundaries, where the professional faces multiple demands. They are paths drawn from technological innovations in Assisted Reproduction, which have placed the psychologist/psychoanalyst in front of new psychological variables. It is science progress producing effects on the participants’ psychological reality, promoting new unconscious representations. Ribeiro (2012) supports that the desire to have a child is filled with unconscious meanings and the experience of infertility seems to reactivate areas of psychological conflicts in those who experience it.

How is the role of this professional outlined, in this moment of the treatment where laymen’s imaginary establishes that “nothing is impossible for science”? Inside a technological apparatus, that at times inhibits participants from visualizing difficulties or the unpredictable.

The modern world points to the confirmation of changes that assign us the task of withdrawing them from a commonplace to start thinking about their implications (Juste *et al*., 2005). Before that, when facing the symptom of infertility, adoption was the answer to having a child. There was a kind of resignation when dealing with infertility and taking on the role of a stepmother/stepfather. New ways of gestating as well as new families start being built, having the Assisted Reproduction Technology as the third parent in the relationship. This intervention exerts effect on sexuality, stressing at times the eroticism of parenthood. It is possible to bear a child without having the slightest intention of an intercourse: it is the “virgin birth syndrome”, according to Lowenkron (2001). The desexualization of motherhood and fatherhood as well as the idealization of these roles is recurrent.

Therefore, we should be careful when interpreting the multiple factors and symptoms present in this process. Tubert (1996) points out that “the couple is a necessary condition but it is not enough for reproduction”. There are anthropological proofs supporting that maternity cannot solely refer to the reproductive potential of the woman or couple. Some peoples from Africa consider reproduction a process triggered by a couple having sexual intercourse, but later on; it evolves through the intervention of an ancestor and other powers of the invisible world. Among the Togo people, for instance, besides the habitual grandparents, a deity named Bomeno - the primary mother - also takes part in reproduction” (Tubert, 1996).

Assisted Reproduction Technology is a universal practice. Today, there are more than eight million individuals registered as being born through these techniques, with a growing demand and many times without its rightful access (Souza *et al*., 2018). In Latin America, the recently published data from the Latin American Registry in its 27^th^ Edition (Zegers-Hochschild *et al*., 2019) report on 75,121 cycles initiated in the year of 2015, resulting in the birth of 18,391 babies until September 2016. In the same period, there were 3,232 egg-freezing cycles reported.

Today, there are new ways to better meet these social demands, for individuals or couples, aiming for a space destined to them and a time to be heard, within the health care team. However, there are different insights, with diversified professional understandings, where the listening and speaking are many times distinct. Whatever was said or not said in the face of the unpredictable, the facts and the experience are understood and “heard” in different ways as well. It is necessary to establish an interface of this “listening” among the participants of a clinical team in order to achieve a unity of praxis.

## OBJECTIVE

To identify the role and possible participation of a psychologist/psychoanalyst inside an Oocyte Pick-up Room and a Recovery Room in an Assisted Reproduction clinic and the implications when attending to patients and participating in the team.

## MATERIALS AND METHODS

We ran a prospective study on the psychological interventions in the OPR-RR in the period from September 2014 to December 2018, performed during the procedures, as well as their effects on the professional team. The visits, most of the time, occurred at the moment of retrieving oocytes, for either IVF/ICSI or gamete freezing, at Fertipraxis Clinic, a private care center, located in Rio de Janeiro, Brazil. The clinical staff comprises medical specialists, who see patients and run ultrasonographic examinations, a psychologist/psychoanalyst, a nurse manager, licensed nurses and embryologists. Besides its own patients, the clinic also receives patients from affiliated physicians who make use of the Center and the Assisted Reproduction laboratories. Seeking a consistency of behavior and continuing education, all the professionals involved take part in the weekly meetings and clinical discussions. All patients sign an Informed Consent Form and the clinic holds a work license issued by ANVISA and is accredited by the Latin American Network of Assisted Reproduction (REDLARA).

In the Oocyte Pick-up Room and in the Recovery Room (OPR-RR), abiding by the Brazilian regulations, according to the Collegiate Board Resolution - RDC no. 23 of the Brazilian Health Regulatory Agency - ANVISA on May 25, 2011 (ANVISA, 2011), the following is performed: follicle aspirations for IVF/ICSI or egg cryopreservation, PESA procedures or testicular biopsies, embryo transfers - not to mention endometrial receptivity array tests - the ERA Test^®^. Every patient, in the first medical appointment, receives an information leaflet where they can have access to the detailed description of each stage of the treatment, in addition they may count on the presence, participation and follow-up meetings performed by the clinic`s psychologist/psychoanalyst. The psychological assistance was performed on the days of aspirations, transfers, PESA procedures and ERA Tests^®^, according to the availability given by the professional, always at the patient’s bedside, before and after procedures, in addition to the follow-up visit in the room where they are performed, in the surgery center.

Before the appointment itself, but after the patient’s identity is verified, the appropriately dressed patient arrives in the recovery room. First, the patient is received by the nursing team and then taken to the recovery room, which had been previously personalized for the patient. The patient is checked for fasting (if needed for the procedure) and vital signs, and the patient’s information on the last exams are confirmed. From then on, the patient waits for the procedure to be performed. At this point, the psychologist/psychoanalyst heads to the recovery room, introduces herself and confirms the purpose of the assistance, which is planned to last for 20 minutes. If there is no standard question planned, many times it is just enough to start by saying “good morning” or asking, “How are you feeling?” The patient speaks to the psychologist/psychoanalyst and the listening starts. Other professionals in the team who later arrive in the room may also introduce themselves, whether it is the patient’s own assistant physician, an aide or the anesthesiologist.

The psychologist/psychoanalyst takes the patient to the operating room, leaving the patient only after the anesthesiologist has confirmed the patient is sedated or staying, depending on the case. After the patient has returned to the room, a new period of support starts, this time in the presence of the partner or a family companion. There is a record of each visit performed and the relevant data from each case is shared directly with the team, through medical charts and reports.

## RESULTS

Results are shown in the Chart.

**Chart:**
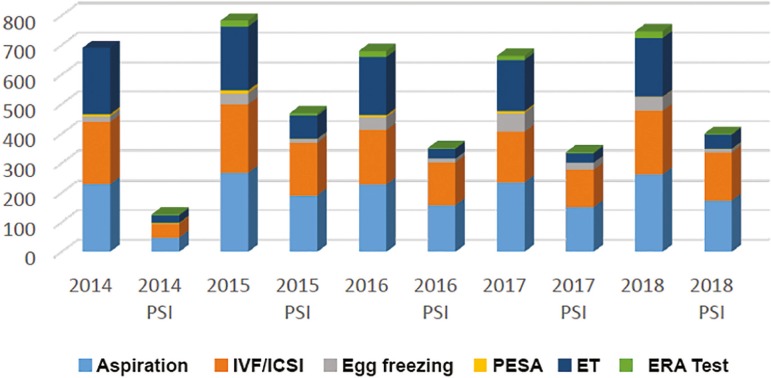
Procedures with and without the intervention of a psychologist/psychoanalyst

All 965 psychological assistance visits performed in the OPR-RR corresponded to 41% of the 2,343 procedures. They were more frequent at the moment of oocyte retrieval for either IVF/ICSI or egg freezing, when the psychologist/psychoanalyst was available in the clinic in 59% of the time (722 cases). The embryo transfers, totaling 1,011 in this period, had psychological assistance in 20% of the times (218 cases). The intrauterine insemination cases were excluded from this study for not being held in a surgical environment.

The recovery room in the Outpatient Surgery Center was identified as one of the spaces for welcoming and listening to patients that is, listening to the anxieties, desires, projects, worries, fears, frustrations, joys and expectations from those who come to the clinic in search of fulfilling the dream of gestating.

The patients’ talks, collected in observations transcribed from what was heard, with dates and types of procedures, were discussed with either the team or the assistant physician. In the same way, either the observations considered pertinent by the psychologist/psychoanalyst, from one individual or the couple, accessible in the electronic medical chart records, lead to subsequent contacts, embryo transfers and even consultations that followed the cycles, successful or not. The grouping of these observation notes is considered in the discussion.

## DISCUSSION

“and at last a way was with difficulty opened up his voice by grief “

Virgil, Aeneid, XI, 151.

We did not find any publication of similar service from an Assisted Reproduction Center, in the period the patients were inside the OPR-RR. The period of this study was influenced by bio-socioeconomic problems, which interfered in the performance of procedures. The year of 2014 presented a lower representation of the presence of a psychologist, because the practice started just in its last trimester (79 out of the 462 annual visits, that is, 17%). The year 2016 had the burden of the Zika problem (Carvalho *et al*., 2016) in Brazil: at the same time that the number of IVF/ICSI procedures dropped 13% in general, for fear and uncertainties in relation to microcephaly and other possible problems for the baby, there was a higher demand for egg freezing (soaring 16%), an alternative to overcome this obstacle. The year 2017 reflected an economic crisis in the country (Cavallini, 2017) and in 2018, there was a return to the pre-Zika environment, with 226 psychological sessions among the 485 procedures in the OPR-RR (47% of the procedures were covered by a psychologist/psychoanalyst).

In the clinical practice, the psychologist/psychoanalyst comes forward to listen to a life story, or many life stories - stories of future parents, grandparents, uncles and aunts or no possible gestational existence. The patient is listened to, so that it becomes possible to identify her symptoms and stories in her speech.

Why “listen” more than “hear”? Hearing is an event; it is something that happens to us as a natural process. Listening, however, is an action; it is something we do consciously. Hearing may be considered more superficial when compared to listening, and listening may correspond to the act of hearing something with thoughtful attention, paying attention to sound.

Understanding that the listening activity performed by the psychologist/psychoanalyst in the OPR-RR may refer to the orientations presented by Freud in his text “Recommendations to Physicians Practicing Psycho-Analysis” (1912), where there are considerations regarding psychoanalytic listening, this kind of listening would consist of not targeting the repair to something specific and keeping the same attention hanging in the face of all that has been heard (Freud, 1912). In this way, we spare ourselves a strain on our attention by only concentrating on what is uttered by the patient. Freud points out that when we deliberately concentrate the attention to a certain degree, we may “never find anything but what we already know”.

Although this recommendation has been made to guide the clinical work of the analyst with the patient, within practice boundaries, it is possible to observe that it can also be applied in the outer practice boundaries. The postmodern era and technological advancement have provided new means of assisting patients (via Internet, Skype, FaceTime, WhatsApp, Emails) preserving the technique of listening or bringing about new ways of listening.

The psychologist in the OPR-RR, exercising the function of assistance, in order to welcome patients and their companions and give attention to their emotional state, heard:

“The emotional distress is horrible in this process”.

“We are an emotional mess inside...the monsters that are asleep, awake”.

The uttered or unuttered words of patients, their demands (what is said about desire), their desires (what is not explicitly said), as well as singularity and subjectivity arise in this process, at a moment when their own lives have stopped to listen to what was urging: do I or do I not want to have a child?

“I am doing my part, but I haven’t been able to get pregnant... I changed clinics just to change the geography. I have already seen a psychologist in the other clinic, before or after procedures but never in the recovery room. I am used to leaving a year to mourn in between post-transfer losses.”

We can then say that the role of the psychologist/psychoanalyst has grown wider and so has the listening activity, enabling new listening moments within the OPR-RR environment. However, we cannot forget that these patients are not going through a process of psychoanalysis or therapy. These patients are in fact being listened/heard to or psychologically accompanied throughout these procedures. Mezan (2002) also indicates that this participation in a multiprofessional team, despite not being similar to the activity performed in an office, would present close ties: “deep inside it is the same thing, the same direct contact with psychological sufferings, defenses, fantasies and transferences”.

We listen to patients’ words that are registered in their life stories. Past, present and future are revealed in so little time in the patients’ talks, showing demands addressed to the welcoming of a listening moment. “... these demands are, above all, a matter of speaking, of resonance when crossing desires, that each individual has his/her particularity and logic, that there is no actual body on one side, and symbolic speaking on the other...together in all act of speaking” (Chatel, 1995).

“I’m empty, things didn’t happen the way I thought they would”

Sometimes the patient talks about infertility as a somatic symptom, where the desire comes across as the impossibility of the body, and at other times, as a psychological symptom, where there is the impossibility of expressing the unconscious desire:

“It hasn’t worked ...I’m being punished because I had abortions...but I don’t want to use a donor’s egg”.

“I don’t intend to have kids, I don’t have this project, I never thought about it, but what if I change my mind some day?”

The Assisted Reproduction technique to be performed will enable the patient to meet or not this demand of having a child or even freeze this demand. We can understand this “freezing of demand”, that is, freezing of the project of having a child, as the proof of how difficult it is to gestate, even after the IVF process. This may even turn into the patient’s explanation to society and to him/herself as being “what was meant to be”, apparently acceptable since “not even fertilization was able to solve the problem”. Negation is used not to face the emptiness that the absence of a positive result may provoke.

Ribeiro (2012) considers that the experience of a psychogenic infertility does not only reactivate psychological conflicts related to the desire of having a child but also, predominantly, reactivates and incites areas of psychological conflicts, that is, areas of unelaborate unconscious conflicts, related to sexuality, conflicting attachments regarding maternity, among other conflicts associated to the identity of gender and Oedipal conflicts. We notice that the symptomology of the patient seems to guide patient to what constitutes him/her through his/her own story and report. The listening of this account worded by an individual that speaks establishes “a clinic of listening”, “a clinic of the individual seized by the listening” (Ansermet, 2003).

“I’m very sensitive. I am cut to the quick. I’m trying to get pregnant for over a year now”.

“I don’t want to sleep; I don’t want to go on. I’ll give up, I’m too anxious.”

At times, in this space, the patient provides the psychologist with some information about his personal or family life or even makes inquiries he had not made before to other members of the team.

“I’d like the psychologist not to accompany me during the procedure, because the psychologist reminds me of why I’m here”.

“I’m going through IVF because of my mother’s encouragement and pressure, she wants to have grandchildren... she came along in all exams...but, I don’t intend to have kids right now, I don’t need this...maybe one day I will”.

“It is hard to be in the patient’s shoes, I have low tolerance to pain. I prefer to be sedated at all times, even in the transfer because I’m a virgin”.

“Can adoption bring any difficulty to get pregnant?...I have always asked myself why my mother gave me up. My adoptive mother died when I was 15”.

“I have been married for 14 years but I don’t want to get pregnant now so I need to freeze my egg...what if in two years I get divorced from this husband. He’s not happy with the idea of freezing an embryo either... my kids would be frozen”.

This experience in the OPR-RR, shared with the clinical team, has decreased anxiety, enabling the welcoming feeling and bringing less pressure on patients about to face procedures. When moving from the recovery room to the operating room, for the scheduled procedure, the patient feels welcomed, which reflects in their emotional state. When the patient talks, he/she too listens to him/herself. This listening experience may promote the elaboration and some kind of transformation in his/her own understanding of this treatment time.

“I loved having a psychologist there; I’ll talk to my own psychologist. I think my psychologist is my age and I do not think she has kids... I imagine this story of mine must move her a lot...figments of my imagination”.

“ I’m crying but it’s a good feeling ...you, here in the operating room, seem to be part of a sci-fi movie in these clothes”.

“I didn’t know there would be a psychologist, that’s good, it soothes, and talking is a relief! My husband has varicocele, he gets hung up about all this, methodical, a Virgo, he thought he had cancer in the testicles, he has few sperms...”

“I’m very insightful, we are feeling great. We were warmly welcomed. There is professionalism without losing love and care in the visit”.

“My husband is azoospermic, we underwent a treatment in another clinic and we were treated as numbers...attention and warm welcome is everything”.

The European Society of Human Reproduction and Embryology - ESHRE (Gameiro *et al*., 2015) recommends that the best practices in Assisted Reproduction go through a multiprofessional team, which includes physicians, embryologists, nurses, psychologists and even the administrative personnel. The main concept lies in the psychosocial care, which optimizes the service, providing well-being to patients, focusing on the psychological and social implications. Reality has proven it true and the team stands positively in relation to the presence of a psychologist/psychoanalyst, who brings new perceptions and the development of the whole art of listening in the team, as in the following testimonials:

“Everything flows more smoothly, it seems things are lighter, patients’ tension reduces”... (physician)

“It makes our job easier, although it is one more person going around in these surroundings” (physician)

“Very interesting, I never thought it could be this way” (resident)

“Ah, that is what a multidisciplinary team is! (resident)

“Great, it helps us” (licensed nurse)

“Some patients even come to us later to tell us things, that they didn’t have the courage to tell even the psychologist...after everything” (licensed nurse)

We noticed that the psychologist/psychoanalyst who is present in the patients and their companions’ assistance space in the OPR-RR in Assisted Reproduction, besides having the clinical experience that the function requires, he/she needs to be capacitated as to how each stage of the procedure occurs, which exams will be performed, which medicines will be prescribed and what their purposes are. This will determine the particularity of this listening activity, facilitating the progress of guidance, the follow-up visit and the welcoming of emotional aspects present in the experience of fertility/infertility. As Foucault warns us (cited by Reis, 2004), “it is not possible to detach theory from experience or method from results, it is necessary to read the deep structures of the visibility in which the field and the look are linked to each other by ‘codes of knowledge”.

## CONCLUSION

The presence of a psychologist/psychoanalyst in the Oocyte Pick-up Room and the Recovery Room in an Assisted Reproduction clinic means an opportunity to listen to patients’ emotions, providing well-being to patients and echoing in the teamwork relationships.
